# Effects of concurrent aerobic and resistance training on vascular health in type 2 diabetes: a systematic review and meta-analysis

**DOI:** 10.3389/fendo.2023.1216962

**Published:** 2023-09-13

**Authors:** Shengju Chen, Kaixiang Zhou, Huayu Shang, Mingyang Du, Linfeng Wu, Yu Chen

**Affiliations:** ^1^ School of Physical Education, Liaoning Normal University, Dalian, China; ^2^ Sports Health College, Chengdu University of Traditional Chinese Medicine, Chengdu, China; ^3^ School of Sports Medicine and Health, Chengdu Sport University, Chengdu, China; ^4^ College of Physical Education, Chongqing University, Chongqing, China

**Keywords:** concurrent training, aerobic and resistance training, type 2 diabetes, vascular structure, vascular function

## Abstract

**Objective:**

To determine the impacts of concurrent aerobic and resistance training on vascular structure (IMT) and function (PWV, FMD, NMD) in type 2 diabetes (T2D).

**Methods:**

The electronic databases PubMed, Web of Science Core Collection, Cochrane Library, Embase, Scopus, CINAHL, and SPORTDiscus were systematically searched for articles on “type 2 diabetes” and “concurrent training” published from inception to August 2, 2022. We included randomized controlled trials that examined the effects of concurrent training versus passive controls on IMT, PWV, FMD and NMD in T2D.

**Results:**

Ten studies were eligible, including a total of 361 participants. For IMT, concurrent training showed a slight decrease by 0.05 mm (95% CI −0.11 to 0.01, p > 0.05). concurrent training induced an overall significant improvement in FMD by 1.47% (95% CI 0.15 to 2.79, p < 0.05) and PWV by 0.66 m/s (95% CI −0.89 to −0.43, p < 0.01) in type 2 diabetics. However, concurrent training seemed to exaggerate the impaired NMD (WMD = −2.30%, 95% CI −4.02 to −0.58, p < 0.05).

**Conclusions:**

Concurrent training is an effective method to improve endothelial function and artery stiffness in T2D. However, within 24 weeks concurrent training exacerbates vascular smooth muscle dysfunction. More research is needed to explore whether longer and/or higher-intensity concurrent training interventions could enhance the vascular structure and smooth muscle function in this population.

**Systematic review registration:**

www.crd.york.ac.uk/PROSPERO/, identifier CRD42022350604.

## 1 Introduction

The global incidence of diabetes is 537 million adults (20−79 years) in 2021, of which 90−95% is type 2 diabetes (T2D) (1). T2D is a chronic condition characterized by persistent hyperglycemia and glucose intolerance. This disease has grown up to be an extremely serious public health problem in the world due to its high mortality, high disability rate and high morbidity. Over time, the hyperglycemia seen in T2D can elevate the risk of both macro- and microvascular complications, resulting in dysfunction across the spectrum of the heart, kidneys, eyes, and vasculature (2), underscoring the imperative of treating T2D to reduce complications, especially those related to micro- and macrovascular disease, intertwined with impaired vascular structure and function.

With the onset of T2D, subclinical manifestations of cardiovascular pathology occur, which in turn may lead to artery stenosis (3). Intima-media thickness (IMT) is an established index of structural change in the artery and increased in T2D (4). Vascular elasticity is an indicator of vascular function. Pulse wave velocity (PWV) is well established arterial stiffness measurement. Patients with T2D have increased PWV when compared with nondiabetic patients. Accelerated arterial stiffness is believed to be related to structural changes within the media of the arterial wall caused by abnormal calcification (5). Impaired endothelial function, long before symptoms, seems to occur in macro- and microvascular in T2D most extensively studied being decreased NO bioavailability (6, 7). Among the subclinical markers, flow-mediated dilation (FMD) measurement is considered the gold standard of endothelial function (8). Simultaneously, vascular smooth muscle (VSM) cells, integral to vascular physiology, exhibit functional impairment along with intricate shifts in the intracellular biomolecular milieu within the context of T2D (9). Nitroglycerin-mediated vasodilation (NMD) is an index of VSM (endothelial-independent) function. Nitrates cause smooth muscle relaxation once they enter the VSM cell through a series of processes that include the bioconversion of nitrate to nitric oxide, stimulation of soluble guanylate cyclase, production of cyclic guanosine monophosphate, and a reduction in cytosolic calcium levels (10). Arterial stiffness is affected not only by structural changes but also by functional variables of the artery (11, 12). The components of impaired vascular structure and function are all associated with cardiovascular mortality and are important markers for evaluating early atherosclerosis development (13). Therefore, interventions to manage and ameliorate vascular health are highly recommended for T2D.

Exercise can effectively reduce the risk of cardiovascular and all-cause mortality and is encouraged by guidelines for T2D. Aerobic exercise (AE) and resistance training (RT) are currently recommended as effective treatments in diabetes management. In this regard, it is well-established that AE and RT induce specific adaptations in vascular homeostasis. The most common explanation for the beneficial effects of AT on the vascular system is that exercise depresses the sympathetic nervous system and the renin-angiotensin system, as well as increases shear stress (14). The improvement of vascular health by RT may be associated with a reduction in risk factors affecting cardiovascular diseases such as body fat mass, HbA1c levels, blood pressure, and inflammatory mediators (15). Concurrent training (CT), combining AE and RT simultaneously in the same training period, is a popular training strategy to impact overall health. Increasing studies have investigated a focus on the outcomes of CT in T2D for the distinct adaptations and health benefits (16, 17). Randomized controlled trials (RCTs) demonstrated that CT showed a higher reduction of hemoglobin A1c, inflammatory markers and fasting glucose than AE or RT alone (18, 19). Furthermore, CT is considered the first choice to improve vascular health (20, 21). However, available studies on this topic in T2D have shown inconsistent and controversial findings. Specifically, some evidence indicated that CT induces beneficial effects on vascular structure and function (17, 22–25), while other studies revealed that CT does not affect vascular function (24–30). Therefore, there is a need for pooled data and meta-analysis to clarify the efficacy of CT for vascular health to draw more comprehensive and robust conclusions so that useful guidelines for T2D can be generated.

Therefore, this systematic review aimed to examine the effects of CT on vascular structure, vascular stiffness, endothelial and VSM function in patients with T2D by incorporating the latest evidence and indicators, and comparison of inter-group differences to test whether CT is beneficial to the vascular complications of type 2 diabetics.

## 2 Materials and methods

This systematic review and meta-analysis was conducted using Preferred Reporting Items for Systematic Reviews and Meta-Analysis guidelines (31) and registered with PROSPERO (Registration ID CRD42022350604), an international prospective registry for systematic reviews.

### 2.1 Search strategy and study selection

A systematic literature search for relevant studies published was conducted in the electronic databases Cochrane Central Register of Controlled Trials (CENTRAL), Web of Science, Scopus, PubMed (including MEDLINE), Cumulative Index to Nursing and Allied Health Literature (CINAHL), SPORTDiscus, US Registry of clinical trials (www.clinicaltrials.gov) from inceptions until August 2022. Potentially relevant key terms (and synonyms searched for by the MeSH database) were collected through experts’ opinions, literature review and included in the electronic databases in different combinations using a Boolean search strategy with the operators “AND”, “OR” and “NOT”: *strength training, resistance training, endurance training, aerobic exercise, vascular function, vascular structure, type 2 diabetes, and concurrent training*

The search syntax can be found in the Electronic **Supplementary Material** (see **Supplementary Table S1**). We additionally track the references of included articles and relevant systematic reviews and meta-analyses to identify potential studies.

### 2.2 Eligibility criteria

Studies eligible for inclusion in this meta-analysis are required to fulfil the following formal criteria: peer-reviewed original research, and full-text availability. In addition, we followed a PICOS (population, intervention, comparator, outcome, study design) approach to select eligibility for inclusion (**Table 1**). Two authors (S.C. and Y.C.) reviewed the titles, abstracts, and/or full-texts for each of the articles identified by the literature search after the removal of duplicates, aiming to determine the eligibility for this meta-analysis. During the study selection process, discrepancies were resolved by discussion with a third author (K.Z. or H.S.).

**Table 1:** Study selection.

Category	Inclusion criteria	Exclusion criteria
Population	T2D, irrespective of sex and level of physical activity	Individuals with major macrovascular complications (coronary artery disease, cerebral vascular disease) or microvascular complications (e.g., nephropathy, neuropathy, retinopathy)
Intervention	Concurrent training interventions (i.e., a combination of AE and RT)	Single-mode training interventions (e.g., single-mode AE or RT); intervention duration ≤ 4 weeks
Comparator	Passive control group	Absence of a control group, active controls
Outcome	Measures of vascular structure and/or function (i.e., IMT, PWV, FMD, NMD)	No reported measures of vascular function and/or structure
Study design	RCTs	Non-RCTs

T2D type 2 diabetes, AE aerobic exercise, RT resistance training, IMT intima-media thickness, PWV pulse wave velocity, FMD flow-mediated dilation, NMD nitrate-mediated dilation, RCT randomized controlled trial.

### 2.3 Risk of bias and quality assessment

Two investigators (S.C. and Y.C.) independently assessed the risk of bias in eligible studies using the Cochrane risk-of-bias tool (RoB2) (32). The tool assesses bias in five domains: randomization process, deviations from intended interventions, missing outcome data, measurement of the outcome and selection of the reported result. Each domain was scored as low risk, some concern, or high risk. The summary score for each study was evaluated as follows (1): low risk of bias, is that all domains were low risk; or (2) some concerns, if ≥ 1 domain was some concerns, but not to be at a high risk of bias for any domain; and (3) high risk of bias, if ≥ 1 domain was at high risk or some concerns for multiple domains in a manner that substantially lowers confidence in the result. Any disagreements that arose were resolved through discussion with a third investigator (K.Z or H.S.).

### 2.4 Data extraction

Two researchers (SC and YC) performed the search and reviewed the results. The source (name of the first author and year of publication), participant characteristics (age, sex, number), training variables (intervention duration, frequency, session, intensity, and total length of intervention) and main outcomes were extracted. In the case of no agreement regarding data extraction, a third author (K.Z.) was consulted for clarification. To compute effect size, baseline and follow-up means and standard deviations (SDs) for measures of vascular structure or function of both the intervention and control groups were extracted. The extracted data were coded as outlined in **Supplementary Table S2**.

For each outcome, two reviewers (S.C. and Y.C.) used the Grading of Recommendations Assessment, Development and Evaluation (GRADE) methodology to assess the certainty of the evidence as described elsewhere (33). GRADE ratings for each outcome started at ‘high’ due to the randomized controlled trial (RCT) design. Downgrading was determined by the factors of risk of bias, inconsistency, indirectness, impression and publication bias (34).

### 2.5 Data synthesis and statistical analysis

For studies reporting interquartile ranges, the standard deviations were obtained using the methods described in Cochrane Handbook for Systematic Reviews (31). For a trial consisting of 2 CT intervention groups, two subgroups were pooled into a single group using the formulae recommended by the Cochrane Collaboration recommendations (31), to avoid bias due to overweighting of individual trials.

The weighted mean differences (WMDs) with 95% CIs were calculated using fixed-effect models, which could provide more conservative results than random-effects models (31). Statistical heterogeneity was evaluated using heterogeneity chi-squared (χ^2^) and I^2^ values. The level of heterogeneity was interpreted according to guidelines from the Cochrane Collaboration: I^2^ values of 25%, 50% and 75% for low, moderate, and high heterogeneity, respectively (35). In addition, publication bias was assessed by generating funnel plots and conducting Egger’s test (36). All the analyses were conducted using Stata v17.0 (STATA Corp., College Station, TX). A 2-side p < 0.05 was considered statistically significant unless otherwise indicated.

## 3 Results

**Figure 1:**
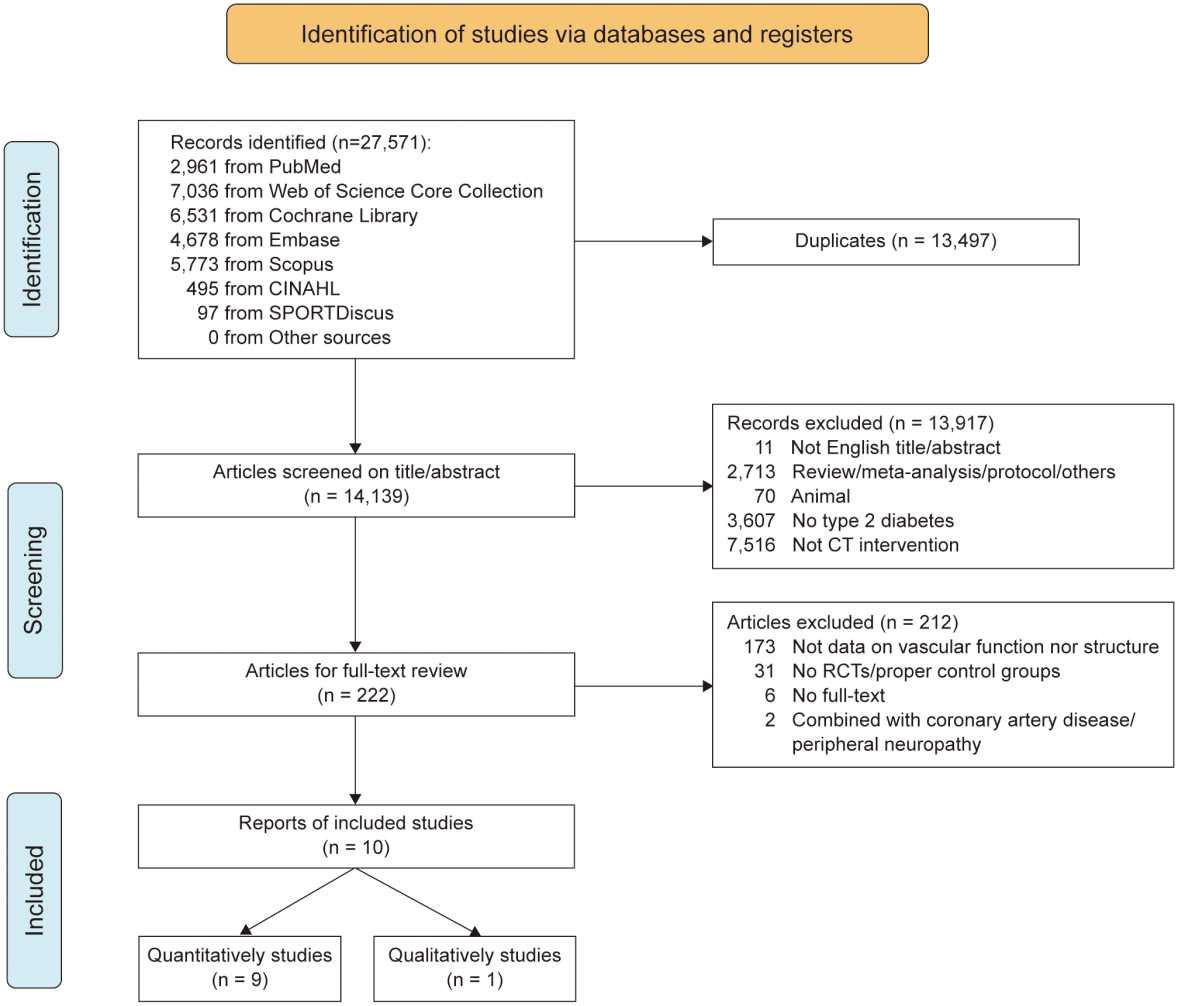
PRISM flow chart illustrating the different phases of the search and study selection. *PRISMA* Preferred Reporting Items for Systematic Reviews and Meta-Analysis.

The results of the systematic search process are illustrated in **Figure 1**. After screening study titles and eliminating duplicates, 14,139 potentially eligible studies were identified. Following the abstract examination, 222 studies remained. After reviewing the full texts, 212 studies were excluded. Finally, 10 studies were eligible for inclusion in this meta-analysis.

### 3.1 Description of the included studies

The 10 eligible studies included an overall sample size of 361 participants, with a mean age ranging from 17 to 65 years (mean 55.84 ± 5.64 years). Out of the included studies, 193 participants received the training intervention and 183 served as control, as one study was a crossover trial (n = 15).

The characteristics of the included studies are summarized in **Table 2**. 2 out of the 10 studies provided data for the effect of CT on vasculature structure (17, 22), 5 studies on artery stiffness (22, 26–29), 4 studies on vascular endothelial function (23–25, 37). 3 studies investigated the effects of CT on VSM function (24, 25, 30). The total length of intervention ranged from 6 to 96 weeks, and training frequency varied from 3 to 6 sessions per week, with CT sessions lasting from 33.1 min to 70 min.

**Table 2:** Characteristics of the included studies.

References	Training moderator variables					Comp	Group	N	Age, year	BMI, kg/m^2^	SBP, mmHg	DBP, mmHg	FBG, mmol/L	HbA_1c_, %	VO_2peak_, ml/kg/min
	idur	tfre	sdur	Intensity RT	Intensity AT										
Maiorana et al^†^ (24).,	8	3	60	Moderate	Moderate-to-high	FMD, NMD	CT	15	52.0 ± 2.0	NA	NA	NA	12.0 ± 1.94	8.5 ± 1.55	23.1 ± 4.65
Maiorana et al^†^ (24),	8	3	60	Moderate	Moderate-to-high	FMD, NMD	Con	15	52.0 ± 2.0	NA	NA	NA	12.0 ± 1.94	8.5 ± 1.55	23.1 ± 4.65
Loimaala et al. (26),	52	4	≥60	Moderate	Moderate-to-high	ap PWV	CT	24	53.6 ± 6.2	29.3 ± 3.7	142.0 ± 17.0	NA	NA	8.2 ± 2.1	31.9 ± 5.1
Loimaala et al. (26),	52	4	≥60	Moderate	Moderate-to-high	ap PWV	Con	25	54.0 ± 5.0	29.8 ± 3.6	145.0 ± 14.0	NA	NA	8.0 ± 1.3	32.6 ± 6.4
Loimaala et al. (27),	96	4	≥60	Moderate	Moderate-to-high	ap PWV	CT	24	53.6 ± 6.2	29.8 ± 3.7	142 ± 13.72	83.0 ± 12.7	NA	8.2 ± 2.1	31.9 ± 5.1
Loimaala et al. (27),	96	4	≥60	Moderate	Moderate-to-high	ap PWV	Con	24	54.0 ± 5.0	29.3 ± 3.6	145 ± 13.72	86.0 ± 11.5	NA	8.0 ± 0.3	32.6 ± 6.4
Okada et al. (25),	12	3-5	60	NA	NA	FMD, NMD	CT	21	61.9 ± 8.6	25.7 ± 3.2	129.0 ± 21.6	74.6 ± 11.6	7.7 ± 2.0	8.5 ± 1.8	22.4 ± 3.2
Okada et al. (25),	12	3-5	60	NA	NA	FMD, NMD	Con	17	64.5 ± 5.9	24.5 ± 2.9	126.6 ± 16.8	73.8 ± 11.8	8.2 ± 1.6	7.9 ± 1.1	22.3 ± 3.7
Lee et al. (28),	6	5-6	60-70	Light	Moderate	ba PWV	CT	23	50.4 ± 7.6	28.6 ± 3.4	125.1 ± 14.0	80.4 ± 10.1	8.1 ± 1.7	7.7 ± 0.7	NA
Lee et al. (28),	6	5-6	60-70	Light	Moderate	ba PWV	Con	12	48.5 ± 7.9	28.1 ± 3.2	131.2 ± 7.8	86.3 ± 4.9	8.8 ± 2.6	8.0 ± 0.9	NA
Barone Gibbs et al. (30),	26	3	>45	Moderate-to-high	light	FMD, NMD	CT	49	58.0 ± 5.0	32.3 ± 5.3	127.0 ± 13.0	72.0 ± 8.0	NA	6.6 ± 1.5	22.7 ± 5.9
Barone Gibbs et al. (30),	26	3	>45	Moderate-to-high	light	FMD, NMD	Con	63	56.0 ± 6.0	33.5 ± 4.3	126.0 ± 13.0	70.0 ± 9.0	NA	6.6 ± 1.4	22.4 ± 5.3
Dobrosielski et al. (29),	26	3	>45	Moderate-to-high	light	cf PWV	CT	70	57.0 ± 6.0	33.0 ± 5.0	126.9 ± 13.4	72.4 ± 9.2	7.6 ± 0.7	6.6 ± 1.7	21.9 ± 5.9
Dobrosielski et al. (29),	26	3	>45	Moderate-to-high	light	cf PWV	Con	70	56.0 ± 6.0	33.6 ± 4.2	126.5 ± 15.9	71.1 ± 9.2	8.3 ± 0.9	6.7 ± 1.7	22.1 ± 5.0
Kadoglou et al. (17),	26	4	45	Moderate-to-high	Light-to-moderate	IMT	CT	22	57.9 ± 6.5	31.9 ± 2.9	138.0 ± 16.0	82.0 ± 12.0	11.2 ± 2.9	8.2 ± 1.0	23.7 ± 5.6
Kadoglou et al. (17),	26	4	45	Moderate-to-high	Light-to-moderate	IMT	Con	24	57.9 ± 7.2	32.1 ± 3.0	135.0 ± 15.0	81.0 ± 11.0	9.9 ± 2.0	7.8 ± 0.8	22.9 ± 3.5
Naylor et al. (23),	12	3	60	Moderate-to-high	Light-to-moderate	FMD	CT	8	17.3 ± 2.3	36.1 ± 11.0	NA	NA	NA	8.8 ± 2.8	25.7 ± 6.8
Naylor et al. (23),	12	3	60	Moderate-to-high	Light-to-moderate	FMD	Con	5	15.3 ± 1.8	30.0 ± 4.9	NA	NA	NA	6.6 ± 0.5	29.9 ± 6.0
Magalhães et al. (22),	52	3	33.1 ± 6.4	Moderate-to-high	moderate	IMT, cf PWV, cd PWV, cr PWV	CT (HIIT)	25	56.7 ± 8.3	30.1 ± 5.7	142.2 ± 18.3	82.6 ± 10.3	NA	4.9 ± 1.2^a^	27.1 ± 6.3
Magalhães et al. (22),	52	3	45.0 ± 7.1	Moderate	moderate	IMT, cf PWV, cd PWV, cr PWV	CT (MCT)	28	59.7 ± 6.5	31.11 ± 5.0	139.9 ± 13.5	82.0 ± 8.8	NA	5.3 ± 2.3^a^	24.1 ± 3.2
Magalhães et al. (22),	52	3	33.1 ± 6.4	Moderate-	moderate	IMT, cf PWV, cd PWV, cr PWV	Con	27	59.0 ± 8.1	30.7 ± 5.0	136.8 ± 13.4	81.2 ± 10.5	NA	5.0 ± 2.1^a^	25.9 ± 5.5

idur intervention duration (weeks), tfre training frequency (sessions/week), sdur session duration (min), N number of participants, FBG fasting blood glucose, HbA_1c_ hemoglobin A1c, VO_2peak_ peak oxygen consumption. NA, not applicable.

^†^Crossover trial.

a Skewed value are presented as median ± inter quartile range.

The approaches for assessing vascular structure (IMT), arterial stiffness (PWV), vascular endothelial function (FMD) and VSM function (NMD) were well described in each individual study except the one by Okada et al. (25). Carotid intima-media thickness was calculated as the average of both sides or single side using carotid ultrasound. PWV is an established metric for assessing vascular stiffness and is measured by different methods which include aortic arch-popliteal PWV (ap PWV), brachial-ankle PWV (ba PWV), carotid-femoral PWV (cf PWV), carotid-radial PWV (cr PWV) and carotid-distal posterior tibial PWV (cd PWV). These values reflect properties of central/aortic and peripheral arterial stiffness for upper and lower limb. Fasting participants were requested to assess FMD through ultrasound, a process wherein the release of NO is prompted by post-ischemic reactive hyperemia, leading to endothelium-dependent vasodilation. A rapid inflation/deflation pneumatic cuff was placed at the forearm in FMD measurement procedures and inflated to 200−250 mmHg to occlude blood flow for 270−300 s. Brachial artery images of diameter were obtained continuously 30 s before and 2−5 min after cuff deflation. After FMD measured, brachial artery diameters were again assessed by sublingual administration of nitroglycerin (0.4−2.5 mg) to calculate NMD using the same methods described for FMD.

### 3.2 Risk of bias assessment and quality of evidence assessment

**Figure 2:**
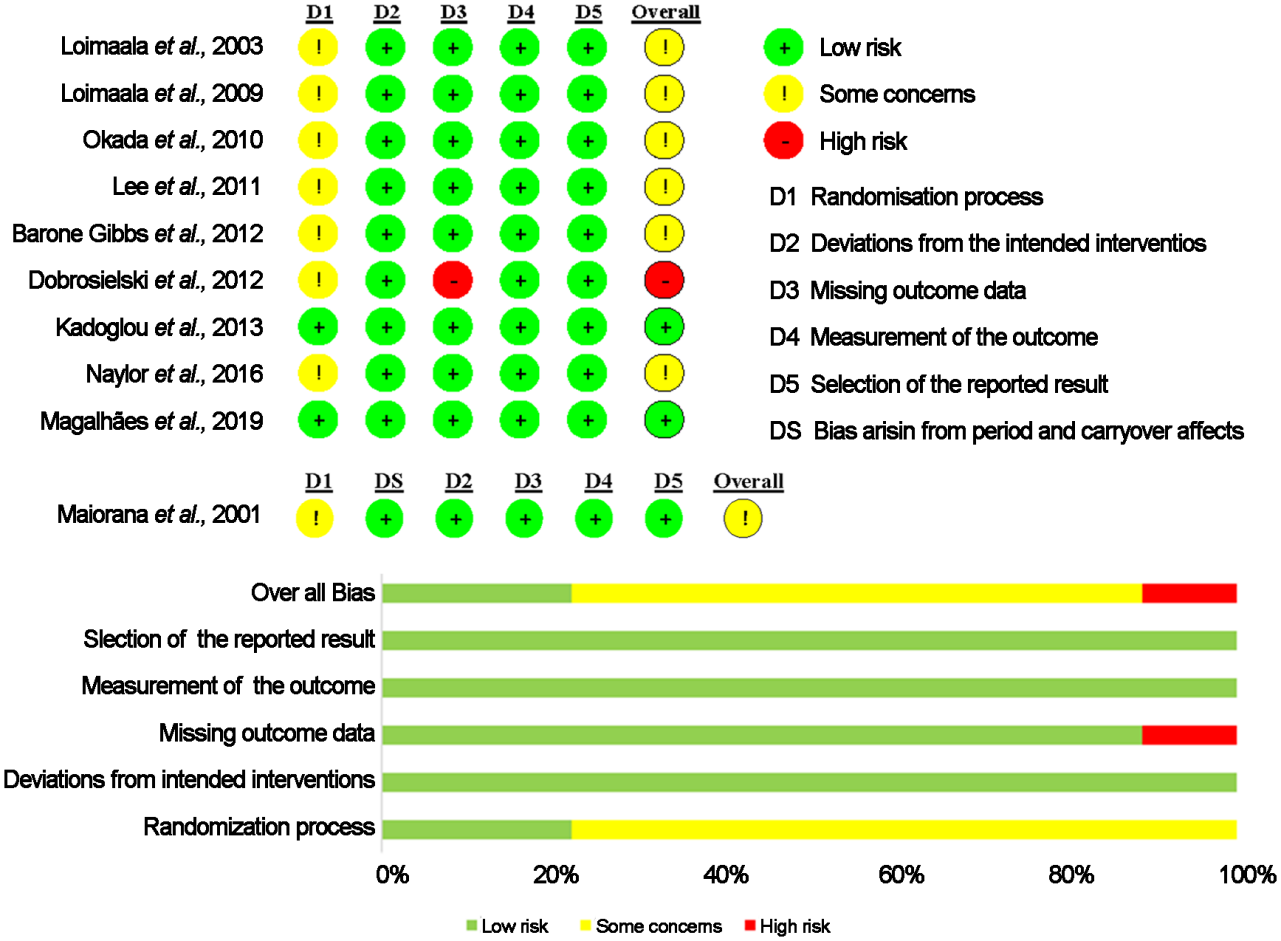
Risk of bias assessment.

The risk of bias estimate is provided in **Figure 2**. Overall, two studies presented an overall low risk of bias across the five domains (17, 22). Only one RCT showed an overall high risk of bias (29). The remaining 7 studies were classified as of some concerns (23–28, 37). Results from the risk of bias assessment using funnel plot asymmetry are displayed in **Supplementary Figures 1A−D**. Egger’s test of the intercept provided no evidence for funnel plot asymmetry and potential publication bias (all p > 0.05).

The GRADE system was used to assess the quality of the evidence. The evaluation results are presented in **Supplementary Table S3**. In Summary, the quality of the evidence was rated as moderate for IMT, PWV, FMD, and NMD due to the small sample size.

### 3.3 Meta-analysis

#### 3.3.1 Effects of CT on vascular structure in T2D

**Figure 3:**
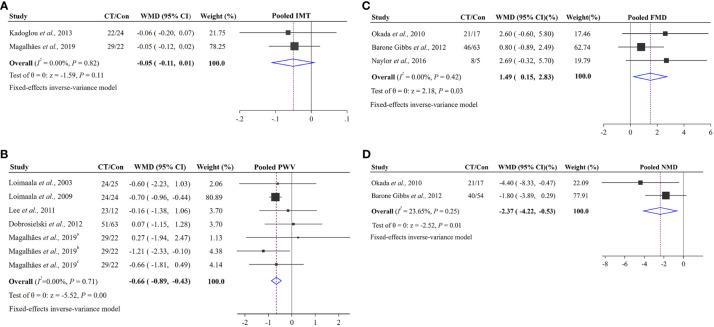
Pooled effects of CT groups vs. control groups on changes in vascular structure and function: **(A)** vascular structure index (IMT); **(B)** vascular function index (PWV); **(C)** vascular function index (FMD); **(D)** vascular function index (NMD). The squares represent the mean difference for each trial. The diamond represents the pooled mean difference across all trials. ^a^ represents cf PWV, ^b^ represents cd PWV, ^c^ represents cr PWV.

Two studies enrolling 97 patients made comparison between the effect of CT on IMT (17, 22). The result of meta-analysis showed that CT could not significantly influence the vascular structure (IMT). The pooled effect size was not significant (WMD = −0.05 95% CI −0.11 to 0.01, p = 0.11) and without the heterogeneity (I^2^ = 0%, p = 0.82; **Figure 3A**).

#### 3.3.2 Effects of CT on vascular function in T2D

Arterial stiffness (PWV), vascular endothelial function (FMD) and smooth muscle function (NMD) were included in 8 RCTs (22, 23, 25–30). As the study by Maiorana et al. was a crossover trial, it would be analyzed in the discussion (38). Seven RCTs enrolling 249 subjects from five studies were analyzed with PWV (22, 26–29). There was an efficacy decrease (WMD = −0.66, 95% CI –0.89 to −0.43, p < 0.001) and low heterogeneity (I^2^ = 0%, p = 0.71; **Figure 3B**) in PWV. Both FMD and NMD were expressed as percentage changes from baseline to maximal dilation. Three studies assessed FMD enrolling 163 T2D patients were included (23, 25, 30). After data pooling, the result of our meta-analysis showed that CT led to a significant increase in FMD by 1.49% (95% CI 0.15% to 2.83%, p = 0.03) without heterogeneity (I^2^ = 0%, p = 0.42; **Figure 3C**). For NMD, two trials enrolling 150 patients with T2D were included (25, 30). Meta-analysis demonstrated that CT could significantly influence the NMD. The pooled effect size was significant (WMD = −2.37%, 95% CI −4.22% to −0.53%, p = 0.01) and with low heterogeneity (I^2^ = 23.65%, p = 0.25; **Figure 3D**).

## 4 Discussion

Our meta-analysis revealed that vascular function adaptation in response to CT might precede structural remodeling in T2D patients. Here, we showed that CT tended to improve the vascular structure (IMT) and significantly enhanced vascular function (PWV, FMD) of T2D patients. However, impaired VSM cell function (NMD) was not alleviated following CT.

In diabetes, thickening and stiffening are early markers of vascular damage (4). Firstly, we investigated the influence of CT on the vascular structure. Results showed that CT tended to reduce IMT by 0.05 mm in T2D patients. IMT has been identified to be strongly and positively associated with the progression of atherosclerosis (4). For every 1mm increase in carotid artery IMT, the risk of diabetic retinopathy increases by 2.9 times (39), but IMT varies according to exercise type and intensity. Kadoglou et al. (17) reported that AE considerably attenuated IMT progression in T2D patients, while CT did not confer any change. However, Magalhães et al. (22) showed that high-intensity AE combined with RT improves IMT more than low-intensity AE combined with RT. This finding is consistent with investigations conducted on obese subjects, where neither aerobic nor anaerobic exercises for 12 weeks yielded noteworthy alterations in IMT (40). Conversely, IMT exhibited a reduction over the same duration of CT (41). Long-term exercise training, on the one hand, alters the shear stress of blood vessels by modulating local blood flow and increases the sensitivity of ion channels in endothelial cells (14). On the other hand, it enhances endogenous antioxidant defense, alters the underlying wall structure, and induces vasodilation, thereby bolstering NO bioavailability (6).

A meta-analysis of 17 longitudinal studies (42) found that each 1m/s increase in resting aortic stiffness corresponds to a 14%, 15%, and 15% increase in the risk of CVD events, CVD mortality, and all-cause mortality, respectively, adjusting for traditional CVD risk factors. Our results showed that arterial stiffness decreased by 0.66 m/s in T2D patients following CT, which is much more than thiazolidinediones (43). On the one hand, this decrease may be related to a function of structural changes in the arteries. Exercise inhibits the development of advanced glycation end products, reduces glucose forms cross-links with collagen proteins within the arteries, and therefore, restores the important balance between elastin and collagen (44). Exercise reduces the generation of reactive oxygen species and inflammation, further promoting the restoration of the structural and functional integrity of vascular wall (44). Alternatively, it could be related to the amelioration of endothelial dysfunction. Endothelial dysfunction can contribute to arterial stiffness in diabetic hypertensive individuals (45). Vasoconstriction in healthy subjects can shift the pressure load bearing toward elastin, unloading stiff collagen. But in individuals with T2DM who have impaired arterial function, vasoconstriction presumably leads to an increase in functional stiffness (46). FMD is a predictor of arterial stiffening (8). Interestingly, a recent meta-analysis of aerobic exercise plus machine-assisted RT failed to provide significant improvement in PWV (0.54 m/s) (47), suggesting that the disturbance of long-term RE to vascular health varies according to population, exercise intensity and frequency. Here, we found that CT had little structural impact, and then we hypothesized that this change in PWV might be related to an increase in endothelial function. It has also been suggested that diabetes-related endothelial dysfunction precedes changes in morphology and structural vessels (48). The increase in FMD (1.49%) observed in T2D subjects might be indicative of restoration of conduit artery endothelial function since a similar extent of FMD impairment has been repeatedly observed in T2D subjects compared with control non-diabetic subjects (45). Accordingly, in addition to the previously reported beneficial effect of AE or RT on FMD in T2D, CT can be considered a valuable strategy to improve endothelial function in this population (21, 49). Moreover, a meta-analysis of 14 prospective studies including 5,547 subjects demonstrated that a 1% increase in FMD was associated with a 13% reduction in the incidence of cardiovascular events (50), thus the 1.47% increase in FMD could indicate a significant effect of exercise interventions on cardiovascular health in T2D patients. Interestingly, as the exercise duration increased, the trend for FMD amelioration was suppressed, which was consistent with the previous review (51). However, as there are fewer included studies, long-term prospective studies are required to determine whether the endothelial function-enhancing effect of exercise is preserved over time and related to a reduced risk of atherosclerosis and CVD in T2D.

In addition to the endothelium, VSM needs to be considered as a potential cause of vascular dysfunction in T2D (52). As T2D progresses, both endothelium-dependent and endothelium-independent vasodilation impairments coexist. However, the pooled effect showed an exacerbating VSM dysfunction (2.37% reduction) in T2D following CT intervention, which was inconsistent with our previous hypothesis. A previous meta-analysis found that exercise, including CT, AT and RT, had no effect on the NMD of T2D for comparing exercise and control groups (51). Furthermore, the time-dependent adaptation in vascular function of exercise in T2D was observed by monitoring FMD and NMD 2-weekly for 8 weeks of exercise. It was found that there was a tendency for FMD to increase and NMD to decrease with time-course, but no significant differences. However, when corrected for endothelium-independent dilation, the FMD/NMD increased across the exercise training programme, resulting in a time-dependent improvement in diabetic endothelial function (53). Some research also supports the notion that exercise-induced adaptions are more pronounced in the endothelial function in arteries compared with VSM function (24, 51). Short-term vigorous exercise exacerbated VSM dysfunction, and reduction in impaired NMD only occurred when exercise intervention lasted longer than 12 weeks (54). With increasing duration, the improvement becomes more evident, which is consistent with our findings that 24 weeks of CT significantly improved the impaired NMD compared with 12 weeks of CT. Nevertheless, this endothelium-independent dysfunction has to be interpreted with caution, given that the endothelium-dependent response includes, at least in part, the function of VSM. The effect of exercise on VSM function may involve the following mechanisms. The mechanical stress stimulation caused by skeletal muscle contraction on blood vessels is related to the type and intensity of exercise, resulting in differences in blood flow shear stress (14), and thereby inducing different degrees of NO release (6). The response to shear stress and the degree of muscle fiber recruitment may be the key to the effectiveness of training. In addition, exercise training, especially RT combined with high-intensity interval aerobics, thickens skeletal muscle capillary thinning due to capillary hemodynamic changes, alters endothelial and VSM cell phenotypes, and enhances glycemic control (19). It is conceivable that longer and/or more intense exercise training programmes could enhance VSM function in T2D patients.

Our results were based on studies that investigated the effect of CT on vascular health, with no emphasis on the mechanistic aspects (e.g., critical pathways of VSM cells signal transduction). Of note, the current research on potential physiological mechanisms has become a hot spot in the field of sports science. As the rate of exercise prescription in the field of chronic disease intervention rises, our findings showed that improvements in vascular function preceded structural remodeling following CT, highlighting the importance of CT in the overall population of T2D. These improvements include the enhancement of endothelial function and reduction of arterial stiffness, but the effective improvement of impaired NMD and remodeling of the vascular structure requires longer intervention.

Despite the effects of CT on structure and vascular function in T2D having been comprehensively investigated, some limitations should be considered when interpreting the results of the analysis. Firstly, only ten studies met the inclusion criteria, one of which was not eligible for the conducted analyses due to a crossover randomized trial (24). These were limited by small sample sizes, for fewer than 50 participants should not be powerful enough. Secondly, few studies reported exercise adherence, which can mask the actual effects of exercise and widen the differences among studies. Finally, some variances existed in the measurements’ parts for PWV. Due to the small sample size, all results of PWV were pooled in our review, so this needs to be considered when applying the results.

## 5 Conclusion

Although individual trials on the effects of CT on vascular function in T2D subjects have shown partially conflicting results, the current meta-analysis suggests that CT is an effective method to improve vascular function (including vascular endothelial function and artery stiffness). Increases of this magnitude would be expected to substantially restore the impaired vascular function commonly found in T2D. Although the relationship between vascular structure and endothelial function in the progression of T2D is still not well understood, the effect of CT on vascular endothelial function precedes structural changes. Future research will be needed to clarify whether longer and/or more intense CT interventions may improve vascular health overall in patients with T2D.

## Data availability statement

The raw data supporting the conclusions of this article will be made available by the authors, without undue reservation.

## Author contributions

SC, YC, KZ, HS, MD and LW made substantial contributions to conception and design; YC designed the study; SC, YC, MD and LW collected the data, SC and YC interpreted the data and wrote the first draft of the manuscript; KZ analyzed the data and contributed to the results; HS reviewed/edited the manuscript. All authors contributed to the article and approved the submitted version.

## Supplementary material

Supplementary Figure 1 Funnel plots of publication bias in IMT, PWV, FMD and NMD. se standard error, WMD weighted mean difference.
