# Supplementary figures and images for: Effects of concurrent aerobic and resistance training on vascular health in type 2 diabetes: a systematic review and meta-analysis
Source: Front Endocrinol (Lausanne). 2023 Sep 13;14:1216962. doi: 10.3389/fendo.2023.1216962 (PMC10534066; doi:10.3389/fendo.2023.1216962)

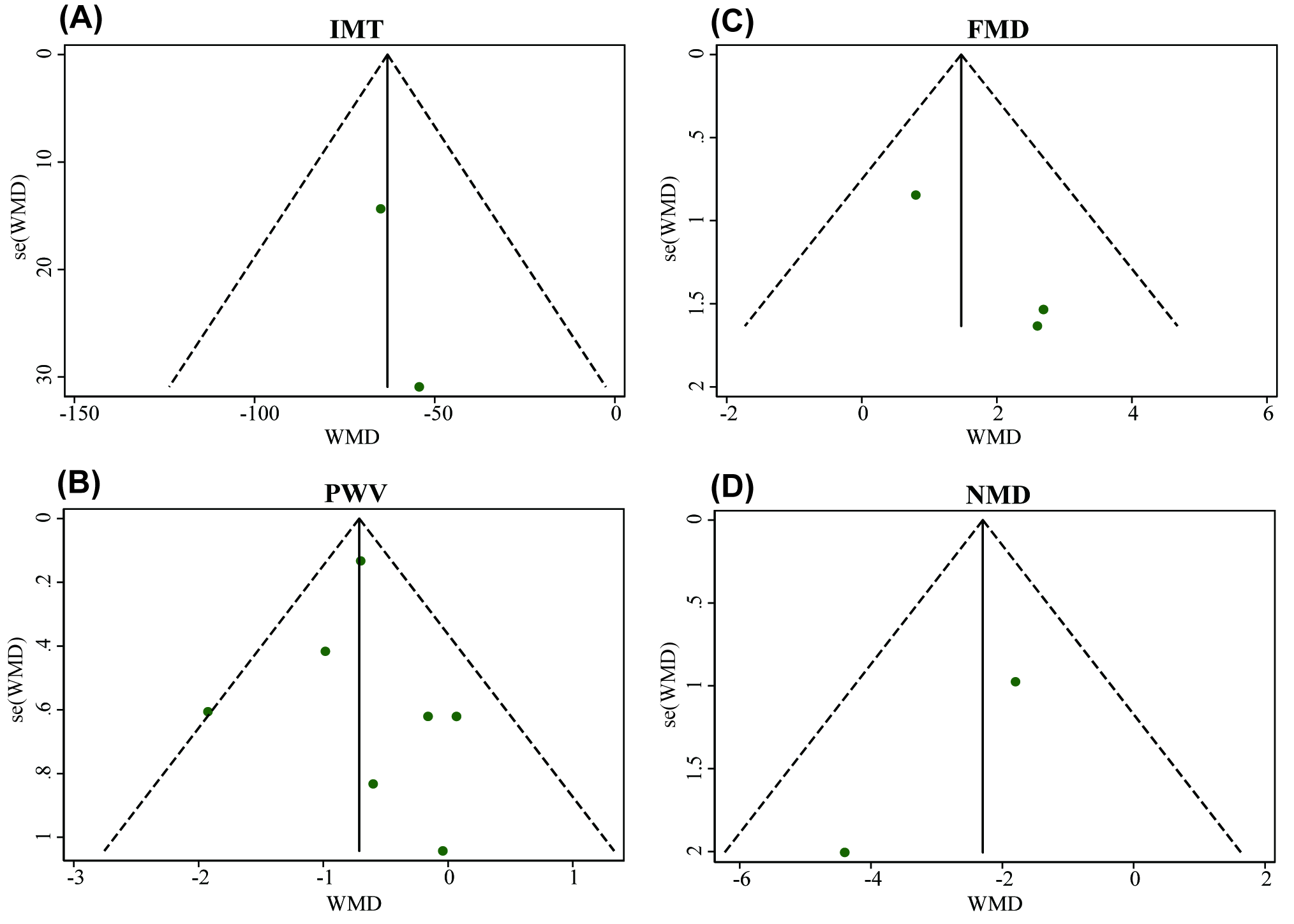

Supplement: Supplementary Figure 1 — Funnel plots of publication bias in IMT, PWV, FMD and NMD. se standard error, WMD weighted mean difference. [file Image_1.tif]
