# Supplementary material for: Effects of concurrent aerobic and resistance training on vascular health in type 2 diabetes: a systematic review and meta-analysis
Source: Front Endocrinol (Lausanne). 2023 Sep 13;14:1216962. doi: 10.3389/fendo.2023.1216962 (PMC10534066; doi:10.3389/fendo.2023.1216962)
Supplement: Supplementary file 2 [file Table_1.docx]

Table S1 The search strategies of databases

| Databases | Search strategies |
| --- | --- |
| PubMed | #1 diabetes mellitus, type 2[MeSH] OR diabetes mellitus, ketosis resistant[Title/Abstract] OR diabetes mellitus, non-insulin dependent[Title/Abstract] OR diabetes mellitus, noninsulin dependent[Title/Abstract] OR non-insulin-dependent diabetes mellitus[Title/Abstract] OR noninsulin-dependent diabetes mellitus[Title/Abstract] OR diabetes mellitus, stable[Title/Abstract] OR stable diabetes mellitus[Title/Abstract] OR diabetes mellitus, type II [Title/Abstract] OR diabetes mellitus, maturity onset[Title/Abstract] OR maturity-onset diabetes[Title/Abstract] OR slow-onset diabetes mellitus[Title/Abstract] OR type 2 diabetes[Title/Abstract] OR diabetes, maturity-onset[Title/Abstract] OR diabetes, type 2[Title/Abstract] OR adult-onset diabetes mellitus[Title/Abstract] OR diabetes mellitus, adult onset[Title/Abstract]  #2 exercise[Title/Abstract] OR physical activity[Title/Abstract] OR training[Title/Abstract] OR concurrent training[Title/Abstract] OR concurrent exercise[Title/Abstract] OR aerobic exercise[Title/Abstract] OR aerobic training[Title/Abstract] OR endurance training[Title/Abstract] OR endurance exercise[Title/Abstract] OR High intensity interval training[Title/Abstract] OR running[Title/Abstract] OR jogging[Title/Abstract] OR swimming[Title/Abstract] OR walking[Title/Abstract] OR cycling[Title/Abstract] OR resistance training[Title/Abstract] OR strength training[Title/Abstract] OR weightlifting training[Title/Abstract] OR strengthening program*[Title/Abstract]  #3 randomized controlled trial [Publication Type] OR randomized controlled trials[MeSH Terms] OR randomized controlled trial [Title/Abstract] OR clinical trials, randomized[Title/Abstract] OR trials, randomized clinical[Title/Abstract] OR controlled clinical trials, randomized[Title/Abstract] OR RCT[Title/Abstract] OR intervention study[Title/Abstract]  #4 animals[mh] NOT humans[mh]  #5 #1 AND #2 AND #3 NOT #4 |
| Web of Science | #1 TS=(diabetes mellitus, type 2 OR diabetes mellitus, ketosis resistant OR diabetes mellitus, non insulin-dependent OR diabetes mellitus, noninsulin-dependent OR diabetes mellitus, stable OR stable diabetes mellitus OR diabetes mellitus, type II OR maturity-onset diabetes OR slow-onset diabetes mellitus OR type 2 diabetes OR diabetes, maturity-onset OR adult-onset diabetes mellitus OR diabetes mellitus, adult-onset)  #2 TS=(exercise OR physical activity OR training OR running OR jogging OR swimming OR walking OR cycling OR strengthening program*)  #3 TS=(randomized controlled trial OR clinical trials, randomized OR RCT OR intervention study)  #4 #1 AND #2 AND #3 |
| Embase | ('non insulin dependent diabetes mellitus'/exp OR 'niddm (non insulin dependent diabetes mellitus)':ti,ab,kw OR 't2dm':ti,ab,kw OR 'adult onset diabetes':ti,ab,kw OR 'adult onset diabetes mellitus':ti,ab,kw OR 'diabetes mellitus type 2':ti,ab,kw OR 'diabetes mellitus type ii':ti,ab,kw OR 'diabetes mellitus, maturity onset':ti,ab,kw OR 'diabetes mellitus, non insulin dependent':ti,ab,kw OR 'diabetes mellitus, non-insulin-dependent':ti,ab,kw OR 'diabetes mellitus, type 2':ti,ab,kw OR 'diabetes mellitus, type ii':ti,ab,kw OR 'diabetes type 2':ti,ab,kw OR 'diabetes type ii':ti,ab,kw OR 'diabetes, adult onset':ti,ab,kw OR 'dm 2':ti,ab,kw OR 'insulin independent diabetes':ti,ab,kw OR 'insulin independent diabetes mellitus':ti,ab,kw OR 'ketosis resistant diabetes mellitus':ti,ab,kw OR 'maturity onset diabetes':ti,ab,kw OR 'maturity onset diabetes mellitus':ti,ab,kw OR 'maturity onset diabetes of the young':ti,ab,kw OR 'niddm':ti,ab,kw OR 'non insulin dependent diabetes':ti,ab,kw OR 'non insulin dependent diabetes mellitus':ti,ab,kw OR 'non-insulin-dependent diabetes mellitus':ti,ab,kw OR 'noninsulin dependent diabetes':ti,ab,kw OR 'noninsulin dependent diabetes mellitus':ti,ab,kw OR 'type 2 diabetes':ti,ab,kw OR 'type 2 diabetes mellitus':ti,ab,kw OR 'type ii diabetes':ti,ab,kw OR 'type ii diabetes mellitus':ti,ab,kw) AND ('exercise'/exp OR 'exercise':ti,ab,kw OR 'exercise training':ti,ab,kw OR 'fitness training':ti,ab,kw OR 'fitness workout':ti,ab,kw OR 'physical conditioning, human':ti,ab,kw OR 'physical exercise':ti,ab,kw OR 'physical work-out':ti,ab,kw OR 'physical workout':ti,ab,kw OR 'physical activity'/exp OR 'activity, physical':ti,ab,kw OR 'physical activity':ti,ab,kw OR 'training'/exp OR 'physical training':ti,ab,kw OR 'training':ti,ab,kw OR 'training course':ti,ab,kw OR 'training program':ti,ab,kw OR 'training programme':ti,ab,kw OR 'training, physical':ti,ab,kw OR 'concurrent training'/exp OR 'concurrent exercise':ti,ab OR 'aerobic exercise'/exp OR 'aerobic dance':ti,ab,kw OR 'aerobic dancing':ti,ab,kw OR 'aerobic exercise':ti,ab,kw OR 'aerobics':ti,ab,kw OR 'aerobics exercise':ti,ab,kw OR 'dancing, aerobic':ti,ab,kw OR 'exercise, aerobic':ti,ab,kw OR 'low impact aerobic exercise':ti,ab,kw OR 'low impact aerobics':ti,ab,kw OR 'step aerobics':ti,ab,kw OR 'aerobic training'/exp OR 'endurance training'/exp OR 'endurance exercise':ti,ab,kw OR 'endurance exercise training':ti,ab,kw OR 'endurance training':ti,ab,kw OR 'endurance workout':ti,ab,kw OR 'endurance-type exercise':ti,ab,kw OR 'endurance-type training':ti,ab,kw OR 'high intensity interval training'/exp OR 'hiie (exercise)':ti,ab,kw OR 'hiit':ti,ab,kw OR 'high intensity interval training':ti,ab,kw OR 'high-intensity intermittent exercise':ti,ab,kw OR 'high-intensity intermittent training':ti,ab,kw OR 'high-intensity interval exercise':ti,ab,kw OR 'high-intensity interval training':ti,ab,kw OR 'intermittent high-intensity training':ti,ab,kw OR 'interval high-intensity training':ti,ab,kw OR 'running'/exp OR 'running':ti,ab,kw OR 'jogging'/exp OR 'jogging':ti,ab,kw OR 'swimming'/exp OR 'swimming':ti,ab,kw OR 'walking'/exp OR 'walking':ti,ab,kw OR 'cycling'/exp OR 'bicycling':ti,ab,kw OR 'cycling':ti,ab,kw OR 'resistance training'/exp OR 'resistance exercise':ti,ab,kw OR 'resistance exercise training':ti,ab,kw OR 'resistance training':ti,ab,kw OR 'resistance-type exercise':ti,ab,kw OR 'resistance-type training':ti,ab,kw OR 'strength training':ti,ab,kw OR 'strength-type exercise':ti,ab,kw OR 'strength-type training':ti,ab,kw OR 'weightlifting training':ti,ab OR 'strengthening program*':ti,ab) AND ('randomized controlled trial'/exp OR 'controlled trial, randomized':ti,ab,kw OR 'randomised controlled study':ti,ab,kw OR 'randomised controlled trial':ti,ab,kw OR 'randomized controlled study':ti,ab,kw OR 'randomized controlled trial':ti,ab,kw OR 'trial, randomized controlled':ti,ab,kw OR 'clinical trials, randomized':ti,ab OR 'trials, randomized clinical':ti,ab OR 'controlled clinical trials, randomized':ti,ab OR rct:ti,ab OR 'intervention study'/exp OR 'intervention studies':ti,ab,kw OR 'intervention study':ti,ab,kw OR 'intervention trial':ti,ab,kw OR 'interventional studies':ti,ab,kw OR 'interventional study':ti,ab,kw OR 'interventional trial':ti,ab,kw) |
| Scopus | #1 TITLE-ABS(“diabetes mellitus, type 2” OR “diabetes mellitus, ketosis resistant” OR “diabetes mellitus, non-insulin-dependent” OR “diabetes mellitus, noninsulin-dependent” OR “non-insulin-dependent diabetes mellitus” OR “noninsulin-dependent diabetes mellitus” OR “diabetes mellitus, stable” OR “stable diabetes mellitus” OR “diabetes mellitus, type II” OR “diabetes mellitus, maturity-onset” OR “maturity-onset diabetes” OR “slow-onset diabetes mellitus” OR “type 2 diabetes” OR “diabetes, maturity-onset” OR “diabetes, type 2” OR “adult-onset diabetes mellitus” OR “diabetes mellitus, adult-onset”)  #2 TITLE-ABS(exercise OR “physical activity” OR training OR “concurrent training” OR “concurrent exercise” OR “aerobic exercise” OR “aerobic training” OR “endurance training” OR “endurance exercise” OR “high-intensity interval training” OR running OR jogging OR swimming OR walking OR cycling OR “resistance training” OR “strength training” OR “weightlifting training” OR “strengthening program*”)  #3 TITLE-ABS(“randomized controlled trial” OR “randomized controlled trial” OR “clinical trials, randomized” OR “trials, randomized clinical” OR “controlled clinical trials, randomized” OR RCT OR “intervention study”)  #4 #1 AND #2 AND #3 |
| SPORTDiscus | S1 SU diabetes mellitus, type 2 OR SU diabetes mellitus, ketosis resistant OR SU diabetes mellitus, non insulin dependent OR SU diabetes mellitus, noninsulin dependent OR SU non-insulin-dependent diabetes mellitus OR SU noninsulin dependent diabetes mellitus OR SU diabetes mellitus, stable OR SU stable diabetes mellitus OR SU diabetes mellitus, type II OR SU diabetes  S2 SU exercise OR SU physical activity OR SU training OR SU concurrent training OR SU concurrent exercise OR SU aerobic exercise OR SU weightlifting training OR SU endurance training OR SU endurance exercise OR SU High intensity interval training OR SU resistance training OR SU strength training  S3 SU running OR SU jogging OR SU swimming OR SU walking OR SU cycling OR SU strengthening program* OR SU aerobic training  S4 S2 OR S3  S5 SU Randomized Controlled Trial OR SU Clinical Trials, Randomized OR SU Trials, Randomized Clinical OR SU Controlled Clinical Trials, Randomized OR SU RCT OR SU intervention study  S6 S1 AND S4 AND S5 |

Table S2 Extracted data of the included studies

| References | Comp | N | Experimental(pre-test) | |  | Experimental  (post-test) | | N | Control  (pre-test) | |  | Control  (post-test) | |
| --- | --- | --- | --- | --- | --- | --- | --- | --- | --- | --- | --- | --- | --- |
|  |  |  | mean | sd |  | mean | sd |  | mean | sd |  | mean | sd |
| Maiorana et al., 2001 | FMD | 15 | NA | NA |  | 5.0^*^ | 1.6^*^ | 15 | NA | NA |  | 1.7^*^ | 1.9^*^ |
| Maiorana et al., 2001 | NMD | 15 | NA | NA |  | 13.7^*^ | 7.8^*^ | 15 | NA | NA |  | 13.1^*^ | 5.8^*^ |
| Loimaala et al., 2003 | ap PWV | 24 | 14.2 | 2.6 |  | 14.8 | 2.3 | 25 | 13.8 | 2.7 |  | 15.0 | 3.7 |
| Loimaala et al., 2009 | ap PWV | 24 | 14.1 | 0.5 |  | 14.7 | 0.4 | 24 | 14.7 | 0.4 |  | 15.4 | 0.4 |
| Okada et al., 2010 | FMD | 21 | 7.3 | 4.7 |  | 10.9 | 6.2 | 17 | 6.4 | 3.6 |  | 7.4 | 5.0 |
| Okada et al., 2010 | NMD | 21 | 14.5 | 6.3 |  | 12.6 | 5.1 | 17 | 9.8 | 5.6 |  | 12.3 | 7.0 |
| Lee et al., 2011 | ba PWV | 23 | 14.2 | 1.9 |  | 13.8 | 1.8 | 12 | 14.0 | 1.9 |  | 13.8 | 1.3 |
| Barone Gibbs et al., 2012 | FMD | 49 | 6.0 | 4.0 |  | 6.6 | 5.0 | 63 | 6.2 | 4.3 |  | 6.0 | 4.2 |
| Barone Gibbs et al., 2012 | NMD | 49 | 10.0 | 5.0 |  | 9.9 | 5.1 | 63 | 9.0 | 4.7 |  | 10.7 | 5.6 |
| Dobrosielski et al., 2012 | cf PWV | 51 | 9.2 | 2.9 |  | 9.3 | 3.3 | 63 | 9.1 | 3.4 |  | 9.1 | 3.6 |
| Kadoglou et al., 2013 | IMT | 22 | 0.8 | 0.2 |  | 0.9 | 0.2 | 24 | 0.8 | 0.2 |  | 0.9 | 0.3 |
| Naylor et al., 2016 | FMD | 8 | 7.6 | 1.2 |  | 9.8 | 1.0 | 5 | 7.8 | 1.0 |  | 7.4 | 1.1 |
| Magalhães et al., 2019 | IMT | 29 | 0.7 | 0.2 |  | 0.7 | 0.1 | 22 | 0.7 | 0.1 |  | 0.7 | 0.1 |
| Magalhães et al., 2019 | cf PWV | 29 | 13.1 | 3.4 |  | 14.0 | 3.7 | 22 | 12.9 | 4.4 |  | 13.5 | 4.1 |
| Magalhães et al., 2019 | cd PWV | 29 | 10.1 | 2.2 |  | 9.8 | 2.1 | 22 | 9.2 | 2.0 |  | 10.1 | 1.8 |
| Magalhães et al., 2019 | cr PWV | 29 | 9.8 | 1.8 |  | 9.2 | 2.0 | 22 | 8.9 | 2.3 |  | 9.0 | 2.1 |

*ap PWV* aortic arch-popliteal artery pulse wave velocity, *ba PWV* brachial-ankle pulse wave velocity, *cf PWV* carotid-femoral pulse wave velocity, *cd PWV* carotid-distal pulse wave velocity, cr PWV carotid-radial pulse wave velocity.

^*^ Change value.

Table S3 GRADE evidence profile

| **Certainty assessment** | | | | | | | **№ of patients** | | **Effect** | **Certainty** | **Importance** |
| --- | --- | --- | --- | --- | --- | --- | --- | --- | --- | --- | --- |
| **№ of studies** | **Study design** | **Risk of bias** | **Inconsistency** | **Indirectness** | **Imprecision** | **Other considerations** | **CT** | **Con** | **Absolute (95% CI)** |  |  |
| **Vascular structure (assessed with: IMT)** | | | | | | | | | | | |
| 2 | randomised trials | not serious | not serious | not serious | serious^a^ | none | 51 | 46 | MD **0.05 lower** (0.11 lower to 0.01 lower) | ⨁⨁⨁◯ Moderate | IMPORTANT |
| **Artery stiffness (assessed with: PWV)** | | | | | | | | | | | |
| 4 | randomised trials | not serious | not serious | not serious | serious^a^ | none | 127 | 122 | MD **0.66 fewer** (0.89 fewer to 0.43 fewer) | ⨁⨁⨁◯ Moderate | CRITICAL |
| **Endothelial function (assessed with: FMD)** | | | | | | | | | | | |
| 3 | randomised trials | not serious | not serious | not serious | serious^a^ | none | 78 | 85 | MD **1.47 higher** (0.15 higher to 2.79 higher) | ⨁⨁⨁◯ Moderate | CRITICAL |
| **Smooth muscle function (assessed with: NMD)** | | | | | | | | | | | |
| 2 | randomised trials | not serious | not serious | not serious | serious^a^ | none | 70 | 80 | MD **2.3 lower** (4.02 lower to 0.58 lower) | ⨁⨁⨁◯ Moderate | IMPORTANT |

*CI* confidence interval, *MD* mean difference, *CT* concurrent exercise, *Con* control.

^a^ Sample size below optimal information size contributing to imprecision which lowers our certainty in effect.
